# Advection-dominated transport past isolated disordered sinks: stepping beyond homogenization

**DOI:** 10.1098/rspa.2022.0032

**Published:** 2022-06

**Authors:** George F. Price, Igor L. Chernyavsky, Oliver E. Jensen

**Affiliations:** ^1^ Department of Mathematics, University of Manchester, Manchester, UK; ^2^ Maternal and Fetal Health Research Centre, University of Manchester, Manchester, UK

**Keywords:** upscaling, homogenization, disordered media, transport

## Abstract

We investigate the transport of a solute past isolated sinks in a bounded domain when advection is dominant over diffusion, evaluating the effectiveness of homogenization approximations when sinks are distributed uniformly randomly in space. Corrections to such approximations can be non-local, non-smooth and non-Gaussian, depending on the physical parameters (a Péclet number Pe, assumed large, and a Damköhler number Da) and the compactness of the sinks. In one spatial dimension, solute distributions develop a staircase structure for large Pe, with corrections being better described with credible intervals than with traditional moments. In two and three dimensions, solute distributions are near-singular at each sink (and regularized by sink size), but their moments can be smooth as a result of ensemble averaging over variable sink locations. We approximate corrections to a homogenization approximation using a moment-expansion method, replacing the Green’s function by its free-space form, and test predictions against simulation. We show how, in two or three dimensions, the leading-order impact of disorder can be captured in a homogenization approximation for the ensemble mean concentration through a modification to Da that grows with diminishing sink size.

## 1. Introduction

Transport processes in many natural systems take place in spatially disordered domains. In many instances, these processes can be adequately described by averaging procedures, Darcy’s law describing flow in random porous media being a well-known example [1]. However, it is important to understand the impact of disorder, particularly in instances where disorder has a significant influence (for example, in explaining breakthrough effects, whereby solute is carried rapidly along a small number of high-flow paths through a random porous medium [2]). The present study contributes to this effort by characterizing the impact of spatial disorder on the uptake of a solute that is advected past distributions of isolated sinks. This problem is loosely motivated by transport of maternal blood in the intervillous space of the human placenta [3] but is posed here in more general terms.

A common assumption that is exploited in order to describe transport in media with complex microstructure is to assume periodicity at the microscale [4–7]. This allows an asymptotic two-scale expansion to be developed, with a unit-cell problem (with periodic boundary conditions) being solved in order to provide a description of slowly varying (homogenized) variables at the macroscale. While this approach has been extended to accommodate slow spatial variation of the microscale field [8–10] and developed for a variety of applications [11–16], it is less adaptable to situations where the microscale exhibits appreciable spatial disorder. Approaches currently adopted in such instances include formal methods of stochastic homogenization [17], spatial averaging techniques [18] or simulations using random microstructures realized within periodic unit cells [19].

A spatially disordered medium can be characterized as a random field with prescribed statistical properties. The ‘forward’ problem that we address here seeks to understand how these properties map to the statistical properties of the concentration field of a solute as it passes through the medium. This map is mediated by physical processes embodied in a partial differential equation (in the present instance, a linear advection-diffusion-reaction equation). The primary question addressed by a homogenization approximation is how to translate the first moment of the sink density to the first moment of the associated concentration field (where first moments are ensemble averages). More refined questions address the impact of spatial disorder, captured in the second moment (covariance) of the sink density, on the mean and covariance of the concentration field. Provided solute fluctuations are bounded in an appropriate sense, these corrections can be evaluated by perturbation around the leading-order homogenization approximation, as we illustrate below, and as demonstrated previously by Dagan [20], Cushman *et al.* [21], Chernyavsky *et al.* [3], Russell *et al.* [22] and Russell & Jensen [23]. If fluctuations become sufficiently large, or if distributions become strongly non-Gaussian, higher moments (or even full probability distributions) of the solute field may need to be evaluated.

Homogenization approximations exploit the separation of lengthscales between the microscale and the macroscale. However, when considering solute uptake at isolated sinks, a further lengthscale needs consideration. The microscale involves two lengthscales, an intersink distance ρ (assumed small compared with the overall size of the domain) and a sink size ς. As ς becomes vanishingly small with respect to ρ, over the shortest lengthscales, diffusion can be expected to dominate advection in the neighbourhood of sinks, and the concentration field can be expected to be described locally by the solution of a diffusion equation in the neighbourhood of a point source. In one dimension, this leads to a concentration field with a staircase structure, with a thin diffusive boundary layer forming upstream of each sink [23]. In two and three spatial dimensions, large solute gradients surround the sink, and the concentration field grows in magnitude proportionally to log⁡(ρ/ς) and ρ/ς, respectively. This effect amplifies fluctuations, as we demonstrate below, and is known to restrict the applicability of homogenization approximations in two and three dimensions [14].

The present study develops an approach initiated by Russell & Jensen [23], who used an iterative method to approximate the effects of disorder in a linear transport problem involving advection, diffusion and solute uptake via first-order kinetics. They considered a spatially one-dimensional problem with uptake taking place at isolated point sinks. They considered parameter ranges for which a steady concentration field can be constructed via a smooth (homogenized) leading-order solution, to which corrections are added that account for the discreteness and disorder of the sink distribution. Corrections are non-local and were evaluated using a Green’s function, sidestepping the assumption of unit-cell periodicity that underlies traditional two-scale homogenization. Russell & Jensen [23] considered a parameter regime in which diffusion was dominant at the intersink distance ρ, allowing the use of Riemann sums to approximate certain sums as integrals. Their approach was constructive: rather than seeking to prove formal convergence, explicit evaluation of the magnitude of corrections allowed domains of validity to be established, and simulation was used to evaluate accuracy. Russell & Jensen [23] demonstrated improved accuracy of corrections to a leading-order homogenization solution evaluated using a Green’s function approach in comparison with classical two-scale asymptotics assuming microscale periodicity. They also compared the magnitude of corrections with solute fields for periodic, normally perturbed and uniformly random sink distributions, each showing distinct dependence on the underlying physical parameters.

Here, we extend this work in four directions, while adopting the same constructive approach: (i) the problem is reformulated to focus on the mapping from statistical moments of the sink distribution to statistical moments of the solute distribution, allowing sink distributions to be represented (for example) as a Gaussian process; (ii) a parameter regime is considered for which advection dominates diffusion over intersink lengthscales, leading to non-smooth concentration profiles; (iii) the study is extended to two and three dimensions, for which the point-sink approximation must be relaxed to allow sinks to have finite size, so that fluctuations remain bounded; (iv) although corrections to a naive homogenization approximation are generally non-local, we show that an essentially local correction to the mean concentration field can be identified when the sink correlation length is sufficiently small, and we evaluate this correction explicitly for sinks distributed uniformly randomly in a two- or three-dimensional domain.

**Figure 1.**
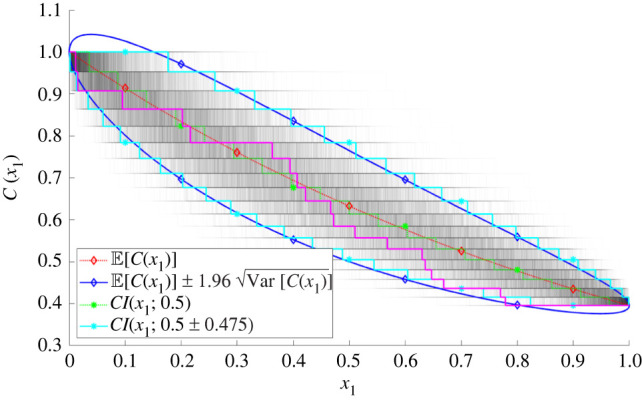
Nineteen point sinks are distributed uniformly randomly along the unit interval, with concentration C(x) falling by a factor $1/(1+S_{1})$ at each one, where $S_{1}=0.05$. From $10^{4}$ realizations of this process, we show: a single realization (solid magenta); the full ensemble of $10^{4}$ concentration profiles (grey); their expectation (E[C(x)], A3, dashed red); Gaussian-based 95% credible intervals ($E[C(x)]\pm 1.96\sqrt{\mathrm{Var}[C(x)]}$, solid blue, using (A 4)); median (CI(x;0.5), dashed green, using (A 8) with r=1/2); cdf 95% credible intervals (CI(x;0.5±0.475), solid cyan, A8). (Online version in colour.)

To set the scene, figure 1 shows a set of realizations of a one-dimensional advection-uptake process (with no solute diffusion). In this example, 19 point sinks are distributed randomly in the domain (0,1), each removing a fixed proportion of the oncoming concentration (which takes the value 1 at the inlet at x=0 and is swept uniformly in the positive x direction). An individual realization (magenta) reveals the staircase structure of a typical one-dimensional concentration field and shows how it deviates appreciably from the discontinuous sample median (green) and the smooth sample mean (red). This example illustrates how the concentration distribution can be non-Gaussian, with credible intervals (cyan) deviating from the equivalent intervals defined by the sample variance (blue) near the source (where concentrations cannot exceed unity) and near the sink (where concentrations cannot fall below $1.05^{-19}\approx 0.396$). This example illustrates how averaging leads to non-smooth concentration fields having smooth statistical moments, even if these must be interpreted cautiously in some circumstances. Expressions for the moments and credible intervals of this simple example are derived in appendix A.

While it is relatively straightforward to make use of an exact Green’s function for a one-dimensional transport problem (satisfying appropriate inlet and outlet boundary conditions), this is less true in two and three dimensions, and the high-dimensional integrals needed to evaluate higher moments quickly become computationally costly. However, when advection dominates diffusion, the free-space Green’s function provides a potentially useful simplification. The Green’s function for advection/diffusion/uptake has a discontinuity in one dimension, a log⁡r singularity in two dimensions and a 1/r singularity in three dimensions, making homogenization feasible for point sinks in one dimension [24] but more challenging in higher dimensions [14]. Accordingly, we consider below isolated sinks of finite width ς, taking them to be distributed uniformly randomly in space. We formulate a transport problem in a domain that is bounded in the advective direction $x_{1}$, assuming a spatially uniform inlet flux at $x_{1}=0$, and assume that sink distributions are statistically uniform over a region that is bounded in the transverse direction. Despite individual realizations having a complex spatial structure, moments typically depend on $x_{1}$ alone, and become smooth as a result of averaging. In the present study, we assume that advection is uniform, ignoring heterogeneity of the flow field or of diffusivity, allowing us to exploit a tractable free-space Green’s function.

In order to capture the effect of disorder within a homogenization approximation, we also adopt a device described by Noetinger *et al.* [25] and exploit the limit in which the correlation length of the covariance of the sink distribution is very small. In the present example, we show that this length is provided by the sink size ς for sinks distributed uniformly randomly in two or three dimensions. This allows us to evaluate an effective uptake parameter ${\mathrm{Da}}_{\mathrm{eff}}$: replacing the dimensionless Damköhler number in the naive homogenized solution with ${\mathrm{Da}}_{\mathrm{eff}}$, we obtain a direct approximation for the mean concentration that quantifies how disorder reduces uptake when sinks are distributed uniformly randomly in two or three dimensions.

The model that we investigate is outlined in §2a, with example simulations presented in §2b. The moments-based expansion is presented in §2c, revealing the critical roles of the Green’s function (§2d) and its singularities in the evaluation of high-dimensional integrals (§2e). The derivation of ${\mathrm{Da}}_{\mathrm{eff}}$ is given in §2f. Predictions are evaluated against simulations in §3.

## 2. Model and methods

### (a) The model problem

We formulate the model in three dimensions, adopting analogues in one and two dimensions when required. Let $D_{3}$ be a domain of thickness L defined such that $x^{*}=(x_{1}^{*},x_{2}^{*},x_{3}^{*})\in D_{3}$ when $x_{1}^{*}\in [0,L]$ and $x_{2}^{*},x_{3}^{*}\in R$. $C^{*}(x^{*};\omega )$, U, D and S represent the (dimensional) solute concentration field, uniform advective velocity in the $x_{1}^{*}$ direction, diffusion coefficient and uptake rate, respectively. Uptake is mediated by a distributed sink function satisfying $1+{\hat{g}}^{*}(x^{*};\omega )\geq 0$, where ${\hat{g}}^{*}$ has zero spatial average. ω denotes that ${\hat{g}}^{*}(x^{*};\omega )$ is a realization drawn from a prescribed distribution, making $C^{*}(x^{*};\omega )$ a random variable.

We prescribe a solute flux q on the plane $x_{1}^{*}=0$, with zero diffusive flux on $x_{1}^{*}=L$ and as $x_{2}^{*},x_{3}^{*}\to \pm \infty$. Defining $x=x^{*}/L$, $\hat{g}(x;\omega )={\hat{g}}^{*}(x^{*};\omega )$ and $C(x;\omega )=C^{*}(x^{*};\omega )/(q/U)$, the dimensionless concentration satisfies the advection–diffusion-uptake equation

2.1*a* $$\nabla_{three-dimensional}^{2}C-\mathrm{Pe}\partial_{x_{1}}C-\mathrm{Da}C(1+\hat{g}(\text{x};\omega ))=0,$$

and boundary conditions

2.1*b* $$(1-{\mathrm{Pe}}^{\;-1}\partial_{x_{1}})C{|}_{x_{1}=0}=1,\;\partial_{x_{1}}C{|}_{x_{1}=1}=0,\;\partial_{x_{2}}C{|}_{x_{2}\to \pm \infty }\to 0,\;\partial_{x_{3}}C{|}_{x_{3}\to \pm \infty }\to 0,$$

where $x_{1}\in [0,1]$, $x_{2},x_{3}\in R$ and $\nabla_{three-dimensional}^{2}\equiv \partial_{x_{1}^{2}}+\partial_{x_{2}^{2}}+\partial_{x_{3}^{2}}$. The Péclet number Pe=UL/D represents the strength of advection to diffusion; the Damköhler number $\mathrm{Da}=SL^{2}/D$ relates the rate of uptake to diffusion. We focus here on the strong-advection regime $\mathrm{Pe}\gg \max(1,\sqrt{\mathrm{Da}})$; of particular interest is the distinguished limit in which Pe⁡/Da=U/SL=O(1), implying a balance between advection and uptake across the whole domain.

Isolated sinks are taken to be of finite size and to occupy a subdomain $D_{3}^{s}$ of $D_{3}$ in which $x_{1}\in [0,1]$ and $x_{2},x_{3}\in [-L_{s},L_{s}]$. Let ρ=1/N be the average inter-sink distance in any direction, where $N\in Z^{+}$ represents the number of sinks per unit length. Let the midpoint of sink locations be represented by $\xi_{i_{3}}=(\xi_{i},\xi_{j},\xi_{k})$, where $i_{3}\in \{i,j,k\}$, i=1,…,N and j,k=−M,…,M with$M=\lfloor L_{s}N\rfloor \in Z$. Thus there are ${\left(2M+1\right)}^{2}/\rho$ sinks in $D_{3}^{s}$ with an average density per unit volume given by $\rho^{-3}$. We define $\hat{g}(x;\omega )$ to be

2.2 $$\hat{g}(\text{x};\omega )=\rho^{3}\sum_{{\text{i}}_{3}}F_{\varsigma }^{\left(3\right)}(\text{x}-\xi_{{\text{i}}_{3}})-1,$$

where $\sum_{i_{3}}\equiv \sum_{i=1}^{N}\sum_{j=-M}^{M}\sum_{k=-M}^{M}$ and $F_{\varsigma }^{\left(3\right)}(x-\xi_{i_{3}})$ is a regularized uptake function with width ς≪1 such that

2.3 $$\int_{D_{3}^{s}}F_{\varsigma }^{\left(3\right)}(\text{x}-\xi_{{\text{i}}_{3}})\;d\xi_{{\text{i}}_{3}}=1.$$

This choice of $F_{\varsigma }^{\left(3\right)}$ ensures $\hat{g}(x;\omega )$ has a spatially averaged density of zero within $D_{3}^{s}$. We assume throughout that isolated sinks have multivariate uniform distribution, such that $\xi_{i}∼U[0,1]$ and $\xi_{j},\xi_{k}∼U[-L_{s},L_{s}]$. Similar definitions of the sink function can be made for a one-dimensional [two-dimensional] domain $D_{1}$ [$D_{2}$], where $F_{\varsigma }^{\left(3\right)}$ is replaced by $F_{\varsigma }^{\left(1\right)}$ [$F_{\varsigma }^{\left(2\right)}$], volumes ($\rho^{3}$) are replaced by distances (ρ) [areas ($\rho^{2}$)] and triple-sums over $i_{3}\in \{i,j,k\}$ are replaced by single- [double-] sums over $i_{1}=i$ [$i_{2}\in \{i,j\}$]. We adopt the Gaussian sink structure function

2.4 $$F_{\varsigma }^{(n)}(\text{x}-{\text{x}}_{{\text{i}}_{n}})=\frac{1}{{\left(2\pi \varsigma^{2}\right)}^{n/2}}\exp\left(-\frac{1}{2\varsigma^{2}}|\text{x}-{\text{x}}_{{\text{i}}_{n}}{|}^{2}\right),$$

where ς remains sufficiently small to satisfy (2.3) and prevent sinks from overlapping, to exponential accuracy. This function is chosen for convenience but could be replaced to model specific applications.

It will be helpful to represent distributions of isolated sinks in terms of their first two statistical moments. As shown in appendix B, uniformly random sinks with Gaussian structure function (2.4) have ensemble mean and covariance

2.5 $$E[\hat{g}]=0,\;K_{\hat{g}}[\text{x},\text{y}]=\rho^{n}F_{\sqrt{2}\varsigma }^{\left(n\right)}(\text{x}-\text{y})-\frac{\rho }{{\left(2M+1\right)}^{n-1}},$$

where $K_{f}[x,y]\equiv K[f(x;\omega ),f(y;\omega )]$ and K represents covariance. An important distinction between one-dimensional and higher-dimensional cases is evident. For n=1, $K_{\hat{g}}$ has a non-local contribution (with N sinks in a one-dimensional domain, finding one sink at a location reduces slightly the chance of finding another elsewhere). However, for n>1, with M→∞, the non-local term vanishes (because the sinks can occupy an arbitrarily wide area or volume within $D_{2}$ or $D_{3}$). The sink density in this case resembles a Gaussian process with square-exponential covariance $\sigma^{2}\exp(-|x-y{|}^{2}/\ell^{2})$, having variance and correlation length given respectively by

2.6 $$\sigma^{2}={\left(\frac{\rho }{2\sqrt{\pi }\varsigma }\right)}^{n},\;\ell =2\varsigma .$$

### (b) Two-dimensional simulations

**Figure 2.**
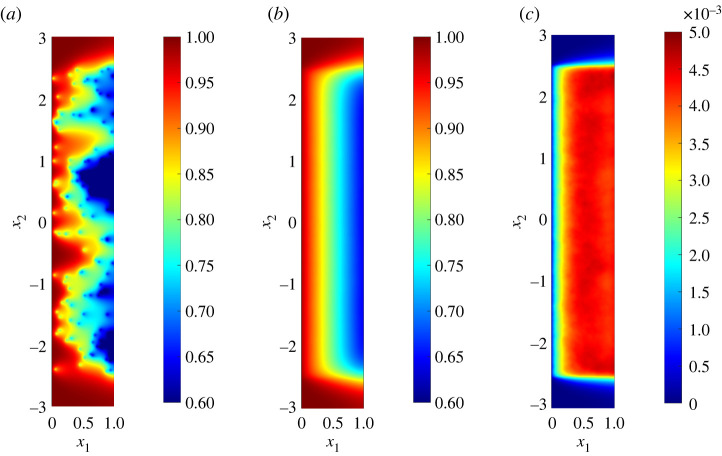
Two-dimensional solute concentration satisfying (2.1) for sinks located uniformly randomly in the domain $D_{2}^{s}=[0,1]\times [-2.5,2.5]$ for ρ=0.2, (Pe,Da)=(20,10) and ς=0.01: (*a*) a single realization; (*b*) sample expectation and (*c*) sample variance, calculated from $10^{4}$ realizations. (Online version in colour.)

Realizations of concentration fields were calculated numerically using a second-order-accurate finite-difference scheme. Representative simulations in two dimensions are shown in figure 2. While an individual realization shows strong disorder, with clear evidence of left-to-right advection (figure 2*a*), the mean concentration field and its variance become smooth and independent of $x_{2}$ when sufficiently far from the boundaries of $D_{2}^{s}$ at $x_{2}=\pm 2.5$ (figure 2*b*,*c*). This arises through a combination of averaging effects and strong advection, which limits the degree of lateral diffusive spread downstream of each sink. We seek approximations of these smooth one-dimensional functions in terms of the sink density ρ, sink width ς and the physical parameters Pe and Da.

### (c) A moments-based expansion

The volume- or ensemble-averaged sink density in (2.1*a*) is unity, making it natural to propose the leading-order homogenized linear and boundary operators associated with (2.1) as $L_{3}\equiv \nabla_{three-dimensional}^{2}-\mathrm{Pe}\partial_{x_{1}}-\mathrm{Da}$ and

B3={(1−(1Pe)∂x1)(⋅)|x1=0, ∂x1(⋅)|x1=1, ∂x2(⋅)|x2→−∞,∂x2(⋅)|x2→∞,1Pe∂x3(⋅)|x3→−∞, ∂x3(⋅)|x3→∞},

respectively. The leading-order homogenized solution $C_{H}(x)$ associated with (2.1) can be found by solving

2.7 $$L_{3}C_{H}(\text{x})=0,\;B_{3}C_{H}(\text{x})=\{1,0,0,0,0,0\}.$$

It is evident that $C_{H}(x)$ depends only on $x_{1}$, being

2.8 $$C_{H}(x_{1})=\frac{\mathrm{Pe}}{\psi (1)}((2\phi -\mathrm{Pe})\;e^{\phi (x_{1}-1)}+(2\phi +\mathrm{Pe})\;e^{\phi (1-x_{1})})\;e^{(\mathrm{Pe}/2)x_{1}},$$

where $\phi \equiv \sqrt{{\mathrm{Pe}}^{\;2}/4+\mathrm{Da}}$ and $\psi (x_{1})\equiv (2\mathrm{Pe}\phi +{\mathrm{Pe}}^{\;2}+2\mathrm{Da})\;e^{\phi x_{1}}+(2\mathrm{Pe}\phi -{\mathrm{Pe}}^{\;2}-2\mathrm{Da})\;e^{-\phi x_{1}}$. In the limit $\mathrm{Pe}\gg \max(1,\sqrt{\mathrm{Da}})$ of interest here, $C_{H}\approx \exp[-\mathrm{Da}x_{1}/\mathrm{Pe}]$, showing how the concentration decays over a lengthscale defined by a balance between uptake and advection. Writing the concentration as

2.9 $$C(\text{x};\omega )=C_{H}(x_{1})+\mathrm{Da}{\hat{C}}_{1}(\text{x};\omega )+{\mathrm{Da}}^{2}{\hat{C}}_{2}(\text{x};\omega )+\ldots ,$$

we construct a solution of (2.1), to be validated *a posteriori*, using the ansatz

2.10*a* $$L_{3}{\hat{C}}_{1}(\text{x};\omega )=\hat{g}(\text{x};\omega )C_{H}(x_{1}),\;B_{3}{\hat{C}}_{1}(\text{x};\omega )=\{0,\ldots ,0\}$$

and

2.10*b* $$L_{3}{\hat{C}}_{2}(\text{x};\omega )=\hat{g}(\text{x};\omega ){\hat{C}}_{1}(\text{x};\omega ),\;B_{3}{\hat{C}}_{2}(\text{x};\omega )=\{0,\ldots ,0\},$$

etc. To invert the linear operators in (2.10), we define $G_{3}(x,x^{\prime })$ to be the associated three-dimensional Green’s function satisfying

2.11 $$L_{3}G_{3}(\text{x},{\text{x}}^{\prime })=\delta (\text{x}-{\text{x}}^{\prime }),\;\text{where}\;B_{3}G_{3}(\text{x},{\text{x}}^{\prime })=\{0,\ldots ,0\}.$$

Applying homogeneous boundary conditions in the $x_{2}$- and $x_{3}$-directions is appropriate as the source term is compact. The Green’s function can then be used to give the corrections

2.12*a* $${\hat{C}}_{1}(\text{x};\omega )=\int_{D_{3}}G_{3}(\text{x},{\text{x}}^{\prime })C_{H}(x_{1}^{\prime })\hat{g}({\text{x}}^{\prime };\omega )\;d{\text{x}}^{\prime }$$

and

2.12*b* $${\hat{C}}_{2}(\text{x};\omega )=\int_{D_{3}}\int_{D_{3}}G_{3}(\text{x},{\text{x}}^{\prime })G_{3}({\text{x}}^{\prime },{\text{x}}^{\prime \prime })C_{H}(x_{1}^{\prime \prime })\hat{g}({\text{x}}^{\prime };\omega )\hat{g}({\text{x}}^{\prime \prime };\omega )\;d{\text{x}}^{\prime }\;d{\text{x}}^{\prime \prime }.$$

We characterize the corrections in terms of their moments evaluated over realizations, specifically

2.13*a* $$\begin{array}{rl} & E[{\hat{C}}_{1}(\text{x};\omega )]=\int_{D_{3}}G_{3}(\text{x},{\text{x}}^{\prime })C_{H}(x_{1}^{\prime })E[\hat{g}({\text{x}}^{\prime };\omega )]\;d{\text{x}}^{\prime }, \end{array}$$

2.13*b* $$\begin{array}{rl} & K_{{\hat{C}}_{1}}[\text{x},\text{y}]=\int_{D_{3}}\int_{D_{3}}G_{3}(\text{x},{\text{x}}^{\prime })C_{H}(x_{1}^{\prime })K_{\hat{g}}[{\text{x}}^{\prime },{\text{y}}^{\prime }]G_{3}(\text{y},{\text{y}}^{\prime })C_{H}(y_{1}^{\prime })\;d{\text{x}}^{\prime }\;d{\text{y}}^{\prime } \end{array}$$

2.13*c* $$\begin{array}{rl} \;\mathrm{and}\; & E[{\hat{C}}_{2}(\text{x};\omega )]=\int_{D_{3}}\int_{D_{3}}G_{3}(\text{x},{\text{x}}^{\prime })G_{3}({\text{x}}^{\prime },{\text{x}}^{\prime \prime })C_{H}(x_{1}^{\prime \prime })E[\hat{g}({\text{x}}^{\prime };\omega )\hat{g}({\text{x}}^{\prime \prime };\omega )]\;d{\text{x}}^{\prime }\;d{\text{x}}^{\prime \prime }. \end{array}$$

This approach extends to n=1,2 dimensions, replacing $D_{3}$ and $G_{3}(x,x^{\prime })$ with $D_{n}$ and $G_{n}(x,x^{\prime }),$ respectively, generalizing the one-dimensional formulation in Russell & Jensen [23]. In higher dimensions, complications emerge due to singularities of $G_{2}$ and $G_{3}$ as $x\to x^{\prime }$ and the high dimensionality of the quadrature.

### (d) The free-space Green’s function

**Figure 3.**
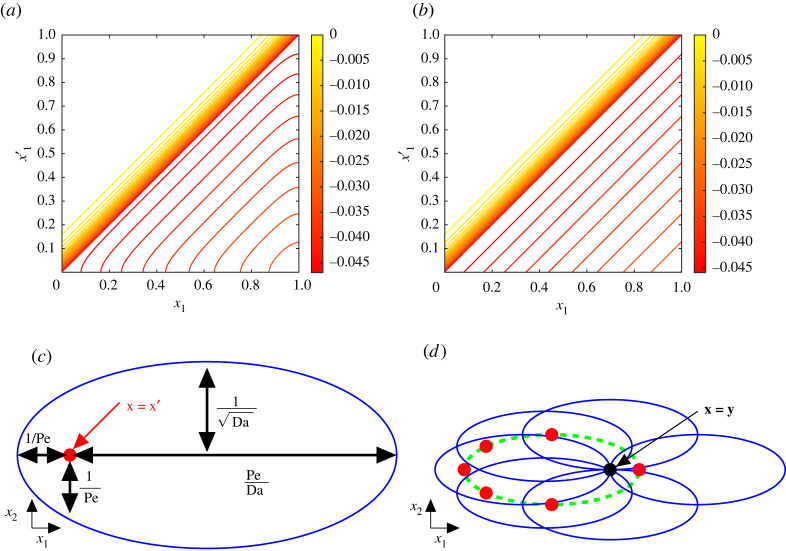
(*a*) Exact $G_{1}(x_{1},x_{1}^{\prime })$ and (*b*) free-space $G_{1}(x_{1}-x_{1}^{\prime })$ Green’s function in one dimension, given by (C 1) and (C 4), respectively, for (Pe,Da)=(20,10). (*c*) Sketch of lengthscales involved in the two-dimensional Green’s function for a sink located at $\text{x}={\text{x}}^{\prime }$ (red dot) and the asymptotic shape of the wake (solid blue), for $\mathrm{Pe}\gg \max(1,\sqrt{\mathrm{Da}})$. (*d*) The asymptotic region of influence (dashed green) about the point x=y (black dot). Sinks located outside of this region will have significantly weaker influence on the concentration at x=y than those inside. Red dots represent sink locations $\text{x}={\text{x}}^{\prime }$ and blue ellipses represent the asymptotic shapes of the wake about each sink. (Online version in colour.)

While the Green’s function in one dimension is straightforward to evaluate (appendix C), it is convenient to instead use the free-space Green’s function $G_{n}(x-x^{\prime })$ for computations in higher dimensions. In three dimensions, this satisfies $L_{3}G_{3}(x-x^{\prime })=\delta (x-x^{\prime })$ and $G_{3}(x)\to 0$ as |x|→∞. $G_{n}$ is given by (C 3): it shares with $G_{n}$ the $\log(\phi |x-x^{\prime }|)$ singularity in two dimensions and $1/|x-x^{\prime }|$ singularity in three dimensions. $G_{n}$ offers a close approximation of $G_{n}$ in the limit $\mathrm{Pe}\gg \max(1,\sqrt{\mathrm{Da}})$, as illustrated for n=1 in figure 3*a*,*b*. This shows a discrepancy between $G_{1}(x_{1},x_{1}^{\prime })$ and $G_{1}(x_{1}-x_{1}^{\prime })$ only within a 1/Pe distance of the outlet in $x_{1}$ and the inlet in $x_{1}^{\prime }$. The identity

2.14 $$\int_{-\infty }^{\infty }\int_{-\infty }^{\infty }G_{3}(\text{x})\;dx_{2}\;dx_{3}=\int_{-\infty }^{\infty }G_{2}(\text{x})\;dx_{2}=G_{1}(x_{1})$$

will allow us to make use of $G_{1}$ later on.

$G(x-x^{\prime })$ denotes the field in the x plane generated by a point sink at $x^{\prime }$. In two dimensions [three dimensions], concentration contours have an approximately elliptical [ellipsoidal] shape, with dimensions illustrated in figure 3*c* when $\mathrm{Pe}\gg \max(1,\sqrt{\mathrm{Da}})$, as explained in appendix C. We can use this structure to identify the asymptotic region of influence associated with a point x, within which sources at $x^{\prime }$ will contribute appreciably to the concentration field at x, as illustrated in figure 3*d*. Strong advection implies that the region of influence is largely upstream of x, while strong uptake ensures that the region is narrow in the direction transverse to the flow. This allows quadrature to be restricted to physically relevant domains.

### (e) Evaluation of moments

Adopting the free-space Green’s function approximation and incorporating the sink moments (2.5), (2.13) becomes

2.15*a* $$\begin{array}{rl} & E[{\hat{C}}_{1}(\text{x};\omega )]=0, \end{array}$$

2.15*b* $$\begin{array}{rl} K_{{\hat{C}}_{1}}[\text{x},\text{y}] & \;=\int_{D_{n}}\int_{D_{n}}G_{n}(\text{x}-{\text{x}}^{\prime })C_{H}(x_{1}^{\prime })G_{n}(\text{y}-{\text{y}}^{\prime })C_{H}(y_{1}^{\prime })\\ & \;\times \left(\rho^{n}F_{\sqrt{2}\varsigma }^{\left(n\right)}({\text{x}}^{\prime }-{\text{y}}^{\prime })-\frac{\rho }{{\left(2M+1\right)}^{n-1}}\right)\;d{\text{x}}^{\prime }\;d{\text{y}}^{\prime } \end{array}$$

2.15*c* $$\begin{array}{rl} \;\mathrm{and}\;E[{\hat{C}}_{2}(\text{x};\omega )] & \;=\int_{D_{n}}\int_{D_{n}}G_{n}(\text{x}-{\text{x}}^{\prime })G_{n}({\text{x}}^{\prime }-{\text{x}}^{\prime \prime })C_{H}(x_{1}^{\prime \prime })\\ & \;\times \left(\rho^{n}F_{\sqrt{2}\varsigma }^{\left(n\right)}({\text{x}}^{\prime }-{\text{x}}^{\prime \prime })-\frac{\rho }{{\left(2M+1\right)}^{n-1}}\right)\;d{\text{x}}^{\prime }\;d{\text{x}}^{\prime \prime }. \end{array}$$

We now consider approximations when the domain width is large ($L_{s}\gg \rho$) and the sink width small (ς→0). To approximate the variance of ${\hat{C}}_{1}$ in this limit, we can replace $F_{\sqrt{2}\varsigma }^{\left(n\right)}$ in (2.15*a*) with an n-dimensional δ-function and note that the second integral in (2.15*b*) can be reduced using (2.14), giving

2.16 $$\begin{array}{rl} {\mathrm{Var}}_{\varsigma \to 0}[{\hat{C}}_{1}(\text{x},\omega )] & \;=\rho^{n}\int_{D_{n}}(G_{n}(\text{x}-{\text{x}}^{\prime })C_{H}{\left(x_{1}^{\prime })\right)}^{2}\;d{\text{x}}^{\prime }\\ & \;\;-\frac{\rho }{{\left(2M+1\right)}^{n-1}}{\left(\int_{D_{1}}G_{1}(x_{1}-x_{1}^{\prime })C_{H}(x_{1}^{\prime })\;dx_{1}^{\prime }\right)}^{2}. \end{array}$$

This reduces the 2n-dimensional integral (2.15*b*) to a cheaper n-dimensional integral (2.16), although some loss of accuracy is anticipated by ignoring the finite sink size.

While (2.14) can also be used to reduce the second integral in (2.15*c*) to one dimension, a δ-function approximation cannot be used for the first integral in $E[{\hat{C}}_{2}]$ because of singularities in $G_{2}$ and $G_{3}$. Instead, we exploit the fact that $F_{\sqrt{2}\varsigma }^{\left(n\right)}(x^{\prime }-x^{\prime \prime })$ is asymptotically small when ς≪1 unless $x^{\prime }$ is within an O(ς) distance of $x^{\prime \prime }$. $C_{H}(x_{1}^{\prime \prime })\approx C_{H}(x_{1}^{\prime })$ over this region while $G_{n}(x^{\prime }-x^{\prime \prime })$ can be approximated by its leading-order singular form. We summarize the results of this calculation (see appendix D), as ς→0 in n dimensions, as

2.17*a* $$\begin{array}{rl} E[{\hat{C}}_{2}(\text{x};\omega )] & \;\approx -\rho^{n}\beta_{n}\int_{D_{1}}G_{1}(x_{1}-x_{1}^{\prime })C_{H}(x_{1}^{\prime })\;dx_{1}^{\prime }\\ & \;\;-\frac{\rho }{{\left(2M+1\right)}^{n-1}}\int_{D_{1}}\int_{D_{1}}G_{1}(x_{1}-x_{1}^{\prime })G_{1}(x_{1}^{\prime }-x_{1}^{\prime \prime })C_{H}(x_{1}^{\prime \prime })\;dx_{1}^{\prime }\;dx_{1}^{\prime \prime }, \end{array}$$

where

2.17*b* $$\beta_{1}=\frac{1}{2\phi },\;\beta_{2}=\frac{1}{4\pi }(\gamma -2\log(2\phi \varsigma ))\;\text{and}\;\beta_{3}=\frac{1}{4\pi^{3/2}\varsigma }$$

and γ is the Euler–Mascheroni constant. The correction in one dimension is independent of the sink size ς as ς→0, whereas in two and three dimensions the correction grows in magnitude as ς becomes asymptotically small. In two and three dimensions, when $L_{s}\gg \rho$, the final terms of $O(\rho /M^{n-1})$ may be neglected and moments become independent of $x_{2}$ and $x_{3}$ when suitably far from boundaries, as illustrated in figure 2*b*,*c*.

Having replaced the exact Green’s function by its free-space form, a further approximation can be obtained by neglecting boundary layers of thickness O(1/Pe) upstream of sinks, evident in figure 3. In one dimension, we adopt the leading-order expressions $C_{H}\approx e^{-(\mathrm{Da}/\mathrm{Pe})x_{1}}$, $G_{1}(x_{1}-x_{1}^{\prime })\approx -(1/\mathrm{Pe})e^{-(\mathrm{Da}/\mathrm{Pe})(x_{1}-x_{1}^{\prime })}H(x_{1}-x_{1}^{\prime })$ for Pe≫1, accounting only for the downstream influence of one sink on another. Direct evaluation of (2.16) and (2.17*a*) gives

2.18 $$\mathrm{Var}[{\hat{C}}_{1}(x_{1},\omega )]\approx \frac{\rho }{{\mathrm{Pe}}^{2}}(x_{1}-x_{1}^{2})e^{-(2\mathrm{Da}/\mathrm{Pe})x_{1}},\;E[{\hat{C}}_{2}(x_{1},\omega )]\approx \frac{\rho }{{\mathrm{Pe}}^{2}}(x_{1}-\frac{1}{2}x_{1}^{2})e^{-(\mathrm{Da}/\mathrm{Pe})x_{1}}.$$

In two dimensions, downstream influence can again be captured approximately by using the far-field approximation (C 7) of $G_{2}$ in the first integrals of (2.16) and (2.17*a*) (taking $\mathrm{Pe}\gg \max(1,\sqrt{\mathrm{Da}})$, ς≪1/Pe and M→∞) to give

2.19 $$\mathrm{Var}[{\hat{C}}_{1}(x_{1},\omega )]\approx \rho^{2}\sqrt{\frac{x_{1}}{8{\mathrm{Pe}}^{3}\pi }}e^{-(2\mathrm{Da}/\mathrm{Pe})x_{1}},\;E[{\hat{C}}_{2}(x_{1},\omega )]\approx \rho^{2}\frac{\log(1/(\mathrm{Pe}\varsigma ))}{2\pi Pe}x_{1}e^{-(\mathrm{Da}/\mathrm{Pe})x_{1}}.$$

In three dimensions, the same approach using (C 10) yields

2.20 $$\mathrm{Var}[{\hat{C}}_{1}(x_{1},\omega )]\approx \frac{\rho^{3}}{8\pi \mathrm{Pe}}\log(x_{1}\lambda \mathrm{Pe})e^{-2(\mathrm{Da}/\mathrm{Pe})x_{1}},\;E[{\hat{C}}_{2}(x,\omega )]\approx \frac{\rho^{3}}{4\pi^{3/2}\varsigma \mathrm{Pe}}x_{1}e^{-(\mathrm{Da}/\mathrm{Pe})x_{1}},$$

where λ=O(1) is a constant that is not determined to this order and the variance expression is not valid near the inlet, when $x_{1}\mathrm{Pe}=O(1)$.

Integrals (2.15)–(2.17) were determined numerically using the solver given in Hosea [26], using adaptive quadrature functions in Matlab. The domain [0,1]×[−3,3] was discretized with 251×1501 points. In one dimension, approximations using the free-space Green’s function were reduced to forms shown in appendix C. The asymptotic region of influence of the two-dimensional Green’s functions (figure 3*d*) was used to identify sufficient domains of integration to ensure convergence.

### (f) Defining the effective Damköhler number

In addition to calculating the mean correction directly via (2.15*c*), we consider how the homogenization problem can be adjusted to capture the leading-order effect of disorder. We seek the constant ${\mathrm{Da}}_{\mathrm{eff}}$ such that the solution of

2.21 $$\nabla_{three-dimensional}^{2}C-\mathrm{Pe}C_{x_{1}}-{\mathrm{Da}}_{\mathrm{eff}}C=0,\;B_{3}C=\{1,0,0,0,0,0\}$$

approximates E[C(x;ω)] to a suitable degree of accuracy. The exact solution of (2.21) is identical to the leading-order homogenized solution given in (2.8) but with Da replaced with ${\mathrm{Da}}_{\mathrm{eff}}$, namely

2.22 $$C_{H}^{UR}(\text{x})=C_{H}^{UR}(x_{1})=\frac{\mathrm{Pe}}{\Psi (1)}((2\Phi -\mathrm{Pe})\;e^{\Phi (x_{1}-1)}+(2\Phi +\mathrm{Pe})\;e^{\Phi (1-x_{1})})\;e^{(\mathrm{Pe}/2)x_{1}},$$

where $\Phi \equiv \sqrt{{\mathrm{Pe}}^{2}/4+{\mathrm{Da}}_{\mathrm{eff}}}$ and $\Psi (x_{1})\equiv (2\mathrm{Pe}\Phi +{\mathrm{Pe}}^{2}+2{\mathrm{Da}}_{\mathrm{eff}})\;e^{\Phi x_{1}}+(2\mathrm{Pe}\Phi -{\mathrm{Pe}}^{2}-2{\mathrm{Da}}_{\mathrm{eff}})e^{-\Phi x_{1}}$. Writing $C(x;\omega )=C_{H}(x_{1})+\hat{C}(x;\omega )$, (2.21) can be rearranged to give $L_{3}\hat{C}(x;\omega )=({\mathrm{Da}}_{\mathrm{eff}}-\mathrm{Da})\times (C_{H}(x_{1})+\hat{C}(x;\omega ))$. Assuming the correction $\hat{C}(x;\omega )$ is small compared with $C_{H}$, the linear operator can be inverted to give

2.23 $$\hat{C}(\text{x})=({\mathrm{Da}}_{\mathrm{eff}}-\mathrm{Da})\int_{D_{3}}G_{3}(\text{x}-{\text{x}}^{\prime })C_{H}(x_{1}^{\prime })\;d{\text{x}}^{\prime }+\ldots ,$$

where the ω notation is dropped as the leading-order correction is deterministic. We then rewrite (2.15*c*) as

2.24 $$E[\hat{C}(\text{x};\omega )]={\mathrm{Da}}^{2}\int_{D_{3}}\int_{D_{3}}G_{3}(\text{x}-{\text{x}}^{\prime })G_{3}({\text{x}}^{\prime }-{\text{x}}^{\prime \prime })K_{\hat{g}}({\text{x}}^{\prime },{\text{x}}^{\prime \prime })C_{H}(x_{1}^{\prime \prime })\;d{\text{x}}^{\prime }\;d{\text{x}}^{\prime \prime }+\ldots .$$

Comparing this with (2.23) gives the approximate relation

2.25 $$\begin{array}{rl} & \;({\mathrm{Da}}_{\mathrm{eff}}-\mathrm{Da})\int_{R^{3}}G_{3}(\text{x}-{\text{x}}^{\prime })C_{H}(x_{1}^{\prime })\;d{\text{x}}^{\prime }\\ & \;\;\approx {\mathrm{Da}}^{2}\int_{R^{3}}\int_{R^{3}}G_{3}(\text{x}-{\text{x}}^{\prime })G_{3}{\hat{K}}_{\hat{g}}({\text{x}}^{\prime }-{\text{x}}^{\prime \prime })C_{H}(x_{1}^{\prime \prime })\;d{\text{x}}^{\prime }\;d{\text{x}}^{\prime \prime }, \end{array}$$

where $G_{3}{\hat{K}}_{\hat{g}}(x^{\prime }-x^{\prime \prime })\equiv G_{3}(x^{\prime }-x^{\prime \prime }){\hat{K}}_{\hat{g}}(x^{\prime }-x^{\prime \prime })$. In (2.25), we have expanded the domain $D_{3}$ to $R^{3}$, a reasonable assumption when sufficiently far from boundaries and the decay lengthscale of $G_{3}{\hat{K}}_{\hat{g}}$ is sufficiently short.

Exploiting the fact that $K_{\hat{g}}(x,y)={\hat{K}}_{\hat{g}}(x-y)$ depends on $x^{\prime }-x^{\prime \prime }$ rather than $x^{\prime }$ and $x^{\prime \prime }$ independently, we can rewrite (2.25) as

2.26 $$({\mathrm{Da}}_{\mathrm{eff}}-\mathrm{Da})G_{3}*C_{H}\approx {\mathrm{Da}}^{2}G_{3}*(G_{3}{\hat{K}}_{\hat{g}})*C_{H},$$

where ∗ denotes convolution. If the decay lengthscale in ${\hat{K}}_{\hat{g}}$ is sufficiently short, then $G_{3}{\hat{K}}_{\hat{g}}$ resembles a δ-function with the appropriate weight and is given by [25]

2.27 $$G_{3}{\hat{K}}_{\hat{g}}(\text{y})\approx \delta (\text{y})\int_{R^{3}}G_{3}{\hat{K}}_{\hat{g}}(\text{x})\;d\text{x}.$$

Fourier transforming (2.26), dividing by the non-zero Fourier transform of $C_{H}$ and applying the inverse transform, we obtain

2.28 $${\mathrm{Da}}_{\mathrm{eff}}\approx \mathrm{Da}\left(1+\mathrm{Da}\int_{R^{3}}G_{3}{\hat{K}}_{\hat{g}}(\text{x})\;d\text{x}\right).$$

As the Green’s function and covariance function are always negative and positive, respectively, ${\mathrm{Da}}_{\mathrm{eff}}$ is smaller than Da, implying that disorder in the sink distributions reduces solute uptake.

For a sink covariance function of the form $\sigma^{2}\exp(-|x-y{|}^{2}/\ell^{2})$, taking ℓ→0 and accounting for the singularity in $G_{2}$ and $G_{3}$, we evaluate (2.28) using methods given in appendix E to give

2.29 $${\mathrm{Da}}_{\mathrm{eff}}\approx \left\{ \begin{array}{ll} \mathrm{Da}\left(1-\sqrt{\pi }\mathrm{Da}\sigma^{2}\ell /(2\phi )\right) & \left(\text{one-dimensional}\right)\\ \mathrm{Da}(1-\frac{1}{4}\mathrm{Da}\sigma^{2}\ell^{2}(\gamma -2\log(\phi \ell ))) & \left(\text{two-dimensional}\right)\\ \mathrm{Da}(1-\frac{1}{2}\mathrm{Da}\sigma^{2}\ell^{2}), & \left(\text{three-dimensional}\right) \end{array}\right.$$

where we have included the corresponding one-dimensional approximation using (2.28). Recall that $\phi =\sqrt{{\mathrm{Pe}}^{2}/4+\mathrm{Da}}$. The correction to Da in (2.29) is proportional to ℓ (one-dimensional), $\ell^{2}\log\ell$ (two-dimensional) and $\ell^{2}$ (three-dimensional), showing how the difference between Da and ${\mathrm{Da}}_{\mathrm{eff}}$ decreases with dimension for fixed variance and fixed correlation length. In one and two dimensions the correction is proportional to 1/ϕ and log⁡(ϕ), respectively, whereas in three dimensions ϕ does not appear in the correction, demonstrating how the impact of advection on the effective uptake decreases as the dimension size increases.

For uniformly random sinks in two and three dimensions letting $L_{s}\to \infty$, we can now use (2.6), noting that the variance depends on sink size, to obtain

2.30 $${\mathrm{Da}}_{\mathrm{eff}}\approx \left\{ \begin{array}{ll} \mathrm{Da}\left(1-\frac{\rho^{2}\mathrm{Da}}{4\pi }(\gamma -2\log(2\phi \varsigma ))\right) & \left(\text{two-dimensional}\right)\\ \mathrm{Da}\left(1-\frac{\rho^{3}\mathrm{Da}}{4\pi^{3/2}\varsigma }\right) & \left(\text{three-dimensional}\right). \end{array}\right.$$

Used in combination with (2.22), $C_{H}^{UR}$ offers a direct estimate for the mean concentration field E[C] for uniformly random sink locations in two and three dimensions, as we illustrate below.

## 3. Results

**Figure 4.**
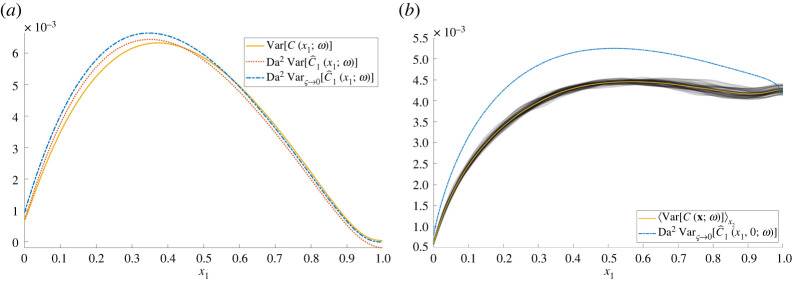
Variance of the concentration for ρ=0.2, (Pe,Da)=(20,10). (*a*) One-dimensional: $\mathrm{Var}[C(x_{1};\omega )]$ (solid) represents the sample variance from $10^{6}$ Monte Carlo realizations, $\mathrm{Var}[\hat{C}(x_{1};\omega )]$ (dotted) is calculated using (2.15*b*) with ς=0.01; ${\mathrm{Var}}_{\varsigma \to 0}[\hat{C}(x_{1};\omega )]$ (dot-dashed) is calculated using (2.16). (*b*) Two-dimensional: the cloud plot (grey) shows the sample variance for $x_{2}=-2,-1.996,\ldots ,2$ from figure 2*c*, the average of these variances over $x_{2}$ [$\langle \mathrm{Var}[C{\left(x;\omega )]\right\rangle }_{x_{2}}$, solid] and the δ-function approximation of the variance from (2.16) [${\mathrm{Var}}_{\varsigma \to 0}[{\hat{C}}_{1}(x_{1},0;\omega )]$, dot-dashed]. Sample variances are calculated from $10^{4}$ Monte Carlo realizations. (Online version in colour.)

The variance of the concentration field in one and two dimensions is illustrated in figure 4. The variance is smooth in both cases, due to strong mixing of sink locations over realizations. In one dimension, because exactly N sinks are encountered along the domain, the concentration at the outlet is strongly constrained (as it was in figure 1), and the variance falls close to zero at the outlet. In two dimensions, this constraint is weaker (N sinks are encountered *on average* between $x_{1}=0$ and $x_{1}=1$), so that the variance remains large at the outlet; (2.19*a*), for example, predicts that the two-dimensional variance is largest at the outlet for 4Da<Pe. The cloud plot in figure 4*b* demonstrates the magnitude of the sampling error from $10^{4}$ two-dimensional simulations, and the independence of the transverse coordinate $x_{2}$ (figure 2*c*).

Figure 4*a* shows how the variance in one dimension predicted by (2.13*b*) matches closely with the sample variance taken from Monte Carlo simulations. In one dimension, the limit ς→0 can be taken straightforwardly, using (2.16), and it provides a good approximation to the sample variance and the full integral (2.15*b*), while overpredicting the predicted variance uniformly. The approximation (2.18*a*), using the leading-order approximation of the free-space Green’s function for Pe≫1, captures the shape of the variance well but overestimating its maximum (predicting 0.0081 at $x_{1}\approx 0.38$ for the chosen parameter values, capturing its $x_{1}$-location well but overestimating its value 0.0063 by almost 30%). In two dimensions, numerical evaluation of (2.15*b*) is expensive so we show only the simplified approximation (2.16), which overestimates the sample variance by approximately 10% (due to neglect of finite sink size) but captures the overall features reasonably well. The cruder prediction (2.19*a*) is also effective: it predicts the maximum variance at $x_{1}=\mathrm{Pe}/(4\mathrm{Da})$ (for Pe<4Da) with value $\rho^{2}{\mathrm{Da}}^{2}/\sqrt{16{\mathrm{Pe}}^{3}\pi e}$; the prediction (0.5,0.0038) underestimates the sample variance 0.0045 by about 15%.

**Figure 5.**
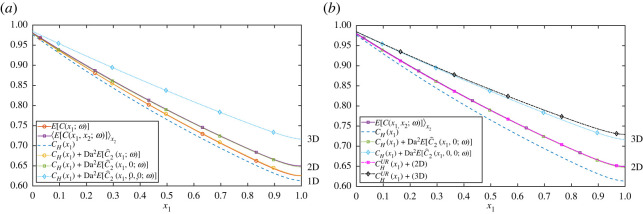
Expected concentrations. Circles, squares and diamonds represent one-dimensional (1D), two-dimensional (2D) and three-dimensional (3D) domains, respectively. (*a*) Dashed and dotted lines represent the leading-order homogenized solution $C_{H}(x_{1})$ plus the approximation ${\mathrm{Da}}^{2}E[{\hat{C}}_{2}(x;\omega )]$ using (2.17*a*). Solid lines represent the sample expectation, using $10^{6}$ realizations (one-dimensional) [$E[{\hat{C}}_{2}(x_{1};\omega )]$] and $10^{4}$ realizations (two-dimensional) with$D_{2}^{s}=[0,1]\times [-2.5,2.5]$, averaging over $x_{2}=-2,-1.996,\ldots ,2$ [$\langle E[{\hat{C}}_{2}{\left(x_{1},x_{2};\omega )]\right\rangle }_{x_{2}}$], for ρ=0.2, ς=0.01 and (Pe,Da)=(20,10). (*b*) As in (*a*), with two- and three-dimensional effective uptake approximations in magenta and black, respectively. $C_{H}^{UR}$ was calculated using (2.30) and (2.22). (Online version in colour.)

Predictions of the ensemble mean concentration field are illustrated in figure 5*a*. $E[{\hat{C}}_{2}(x,\omega )]$ is a smooth function of $x_{1}$, given by (2.17*a*), and agrees well with the sample mean in one and two dimensions (stochastic simulations in three dimensions were not undertaken). The correction compensates for the leading-order homogenized solution over-predicting uptake. The corrections grow with dimension, particularly through the factors $\beta_{n}$ from (2.17*b*) as ς→0. In two and three dimensions when taking the limit M≫ρ (i.e. $L_{s}$ is asymptotically large), $E[{\hat{C}}_{2}(x;\omega )]$ can be simplified as the second integral becomes asymptotically small. Therefore, the computational expense of calculating the correction is further reduced to solving one simple one-dimensional integral. The simpler estimate (2.18*b*) places the maximum one-dimensional correction within the domain (but downstream of the maximum variance), of $O(\rho {\mathrm{Da}}^{2}/{\mathrm{Pe}}^{2})$. The two- and three-dimensional estimates (2.19*b,c*) place the maximum correction within the domain for Da>Pe, but at the outlet otherwise (as in figure 5), although they do not capture the weak boundary layer near $x_{1}=1$ evident in the figure.

The mean concentration in two and three dimensions predicted using the ${\mathrm{Da}}_{\mathrm{eff}}$ approximation (2.30) is shown in figure 5*b*. The correction to $C_{H}$ grows with dimension, as expected, due to the increasingly large concentration fluctuations near each (regularized) sink. The approximation provides close agreement to Monte Carlo sampling in two dimensions, and to the prediction (2.17*a*) in two and three dimensions. (Monte Carlo simulations in three dimensions were not attempted.)

## 4. Discussion

This study has characterized the impact of spatial disorder on a transport process described by a linear advection/diffusion/uptake equation, assuming a uniformly random distribution of isolated sinks. Using a leading-order homogenization approximation (2.7), (2.8) as a starting point, corrections were computed that describe the likely size of solute fluctuations around a mean field in a particular realization, and the correction due to disorder when evaluating the ensemble mean concentration. Bearing in mind the limitations of using statistical moments to characterize non-Gaussian concentration fields (figure 1), we used a moments-based expansion to relate the mean and covariance of the sink distribution to the mean and covariance of the solute field (2.13). The first two moments of the sink distribution, when sinks are distributed uniformly randomly (2.5), show an important distinction between one dimension and higher dimensions, namely that in a sufficiently wide domain in two and three dimensions the correlation length of the sink covariance is set by the sink width (2.6). Simulations (figure 2) reveal the multi-scale nature of the problem: despite large concentration fluctuations in the neighbourhood of individual sinks in an individual realization, ensemble averaging leads to smooth moments of the solute distribution with primary dependence only on a single spatial coordinate. Nevertheless, moments demand calculation of high-dimensional integrals, which we simplified by replacing the exact Green’s function with its (explicit) free-space form, confining quadrature to appropriate regions of influence (figure 3*d*), replacing the regularized sink distribution (where possible) with its δ-function approximation, and integrating over lateral dimensions using identities such as (2.14). This allowed accurate predictions of concentration means (figure 5*a*) in one and two dimensions, and of variance in one dimension (figure 4); the over-prediction of solute variance in two dimensions would likely be corrected by use of the regularized sink distribution, albeit using more expensive quadrature. Cruder analytical estimates (2.18), (2.19), (2.20) were achieved by neglecting any upstream influence of one sink on another.

For vanishingly small sinks (the limit ς→0), concentration fields are discontinuous in one dimension (figure 1), and have log⁡(1/ς) and 1/ς singularities in two and three dimensions, respectively. These appear both in corrections to the ensemble-averaged mean concentration (2.17) and in the effective Damköhler number (2.30) that can be used in a modified homogenization approximation in two and three dimensions. The latter approximation cannot be applied for uniformly random sinks in one dimension, because the sink locations are correlated over the whole domain; however it can be applied when sinks are described by a Gaussian process with sufficiently short correlation length (2.29). For sink distributions of fixed variance, the impact of disorder falls as the sink correlation length vanishes (2.29); however, for uniformly random sinks in two and three dimensions the variance of the equivalent Gaussian process rises as ς falls (2.6), contributing to the reduction in uptake captured in (2.30). As reported by Russell & Jensen [23] and Price [27], mean correctors derived assuming periodic sink distributions show different dependence on parameters to those reported in (2.18*b*)–(2.20*b*). For example, considering the expansion (2.9), the dominant corrector in the deterministic periodic problem appears at O(Da) and shares the wavelength of the microstructure, whereas the dominant correction in the uniformly random case is stochastic with smooth variance ((2.18*a*)–(2.20*a*), figure 4) with the mean correction appearing at $O({\mathrm{Da}}^{2})$ (figure 5).

This study has a number of obvious extensions, prominent among which is consideration of other types of spatial disorder. For flow in porous media, one expects the flow field to have disorder that correlates appreciably with the disorder in the sink distribution [28]. The present perturbative approach provides a route for understanding the contributions of flow, sinks and their combination to solute distributions, and it will be interesting to evaluate the present approach against predictions of existing studies of reactive transport in porous media relying either on periodicity assumptions [4,6,7] or averaging procedures [18]. Other obvious factors to consider include unsteady effects, variability in sink strength (considered in one dimension by [22]) and the impact of a nonlinear uptake kinetics (considered by Dalwadi & King [12] using two-scale homogenization). As demonstrated by Chernyavsky *et al.* [3] and others, the statistical properties of the underlying spatial disorder interact with the physical lengthscales associated with transport processes to give a range of possible outcomes. The present study illustrates some of the challenges in stepping away from traditional two-scale approaches towards non-local calculations, drawing attention to the need for efficient schemes for high-dimensional quadrature in order to characterize uncertainty.

We can revisit the expansion (2.9) and use evidence that terms $\mathrm{Da}{\hat{C}}_{1}$ or ${\mathrm{Da}}^{2}{\hat{C}}_{2}$ become comparable in magnitude with $C_{H}$ as evidence of the breakdown of a homogenization approximation. In one dimension, based on the estimates in (2.18), the restriction $\mathrm{Pe}\gg \max(1,\sqrt{\mathrm{Da}})$ must be extended to $\mathrm{Pe}\gg \max(1,\sqrt{\mathrm{Da}},\rho^{1/2}\mathrm{Da})$, which holds along the distinguished limit Pe∼Da for arbitrarily large Pe. The parameter ${\mathrm{Da}}^{2}\rho /\mathrm{Pe}$, measuring the magnitude (relative to $C_{H}$) of the fluctuation variance and the correction to the mean, takes the value 0.05 in figure 1 (with Pe→∞, but with $\mathrm{Da}\rho^{1/2}/\mathrm{Pe}=S_{1}/\rho^{1/2}$; see appendix A) and figures 4*a* and 5*a*. In these examples, fluctuations with standard deviation of order 20% dominate the correction to the mean, of order 5%. In two and three dimensions, however, the range of validity of the approximation is reduced and the correction to the mean (that grows with diminishing sink size) overtakes the fluctuations as the dominant correction. In three dimensions, we require $\mathrm{Pe}\gg \max(1,\sqrt{\mathrm{Da}},{\mathrm{Da}}^{2}\rho^{3}/\varsigma )$ (for $\rho^{3}\ll \varsigma \ll \rho \ll 1$), which confines the distinguished limit to $1\ll \mathrm{Pe}∼\mathrm{Da}\ll \varsigma /\rho^{3}$. The example shown in figure 5 has $\varsigma /\rho^{3}=1.25$: as the figure indicates, the predicted correction to the mean is sufficiently large to call into question the validity of the homogenization approximation in this case. In two dimensions, the constraint on the distinguished limit is $1\ll \mathrm{Pe}∼\mathrm{Da}\ll 1/(\rho^{2}\log(\rho^{2}/\varsigma ))$: the example in figures 2, 4*b* and 5*a* with $\rho^{-2}=25$ therefore sits at this upper threshold, although the predicted corrections are still effective.

## Data Availability

Matlab code: https://doi.org/10.6084/m9.figshare.19706515.

## Appendix A. One-dimensional concentration profiles with zero diffusion

Let $\xi_{i}$ (i=1,…,N) denote point sink locations, distributed as order statistics $U_{j:N}$ taken from a uniform distribution U∼U(0,1) with probability density function (pdf) $\pi_{U}(x)=1$ for 0≤x≤1 and zero otherwise. Each sink location follows a Beta distribution [29] such that $\xi_{j}∼\beta (j,N-j+1)$, where j=1,…,N. Here $\beta (x,y)\equiv t^{x-1}{\left(1-t\right)}^{y-1}/B(x,y)$, where B(x,y)≡Γ(x)Γ(y)/Γ(x+y). The cumulative distribution function (cdf) $F_{\xi_{j}}(x)=P(\xi_{j}\leq x)$ is given by the regularized incomplete beta function

A 1 $$F_{\xi_{j}}(x)=I_{x}(j,N-j+1)=\frac{\int_{0}^{x}t^{j-1}{\left(1-t\right)}^{N-j}dt}{B(j,N-j+1)}.$$

The one-dimensional concentration distribution that falls by a factor $1/(1+S_{1})$ at each sink from its inlet value $C_{0}=1$ (figure 1) satisfies

A 2 $$C(x)=C_{0}-S_{1}\sum_{j=1}^{N}C_{j}H(x-\xi_{j}),\;C_{j}\equiv {\left(1+S_{1}\right)}^{-j}C_{0}.$$

(This problem can be defined as a limit of the one-dimensional form of (2.1), with Pe→∞ taking$S_{1}=\mathrm{Da}\rho /\mathrm{Pe}$ with Da⁡/Pe=O(1).) The probability of being at concentration $C_{j}$ for some given x is $P(C_{j};x)=P(\xi_{j}<x<\xi_{j+1})=F_{\xi_{j}}(x)-F_{\xi_{j+1}}(x)$ for j=1,…,N−1, with $P(C_{0};x)=P(\xi_{1}>x)=1-F_{\xi_{1}}(x)$, $P(C_{N};x)=P(\xi_{N}<x)=F_{\xi_{N}}(x)$. Therefore, the expectation $E[C(x)]=C_{0}P(C_{0};x)+\cdots +C_{N}P(C_{N};x)$ becomes

A 3 $$\begin{array}{rl} E[C(x)] & \;=C_{0}(1-F_{\xi_{1}}(x))+\sum_{j=1}^{N-1}C_{j}(F_{\xi_{j}}(x)-F_{\xi_{j+1}}(x))+C_{N}F_{\xi_{N}}(x),\\ & \;=C_{0}+\sum_{j=1}^{N}(C_{j}-C_{j-1})F_{\xi_{j}}(x)=1-S_{1}\sum_{j=1}^{N}\frac{I_{x}(j,N-j+1)}{{\left(1+S_{1}\right)}^{j}}. \end{array}$$

The variance $\mathrm{Var}[C(x)]=\sum_{i=0}^{N}C_{i}^{2}P(C_{i};x)-{\left(\sum_{i=0}^{N}C_{i}P(C_{i};x)\right)}^{2}$ satisfies

A 4 $$\begin{array}{rl} \mathrm{Var}[C(x)] & \;={\left(C_{0}\right)}^{2}+\sum_{j=1}^{N}({\left(C_{j}\right)}^{2}-{\left(C_{j-1}\right)}^{2})F_{\xi_{j}}(x)-{\left(C_{0}+\sum_{j=1}^{N}(C_{j}-C_{j-1})F_{\xi_{j}}(x)\right)}^{2}\\ & \;=\sum_{j=1}^{N}\left(C_{j}+C_{j-1}-2C_{0}-\sum_{i=1}^{N}(C_{i}-C_{i-1})F_{\xi_{i}}(x)\right)(C_{j}-C_{j-1})F_{\xi_{j}}(x) \end{array}$$

A 5 $$\begin{array}{rl} & \;=S_{1}\sum_{j=1}^{N}(2-\frac{\left(2+S_{1}\right)}{{\left(1+S_{1}\right)}^{j}}-S_{1}\sum_{i=1}^{N}\frac{I_{x}(i,N-i+1)}{{\left(1+S_{1}\right)}^{i}})\frac{I_{\varepsilon x}(j,N-j+1)}{{\left(1+S_{1}\right)}^{j}}. \end{array}$$

E[C(x)] and Var[C(x)] are plotted in figure 1 using (A 1).

The cdf of the concentration $C_{j}$ is given by

A 6 $$F_{C_{j}}(C)=P(C_{j}\leq C(x);x)=P(\xi_{j}>x)=1-F_{\xi_{j}}(x)\;(j=1,\ldots ,N).$$

Let the cdf take a value $F_{C_{j}}(C)=r$. Then (A 6) can be inverted to give the corresponding sink locations as

A 7 $${\overset{˘}{\xi }}_{j}=F_{\xi_{j}}^{-1}(1-r)=\varepsilon^{-1}I_{r}^{-1}(j,N-j+1)\;(j=1,\ldots ,N).$$

We can therefore use (A 2) to find the cdf credible intervals as

A 8 $$CI(x;r)=C_{0}-S_{1}C_{0}\sum_{j=1}^{N}\frac{H(x-I_{r}^{-1}(j,N-j+1))}{{\left(1+S_{1}\right)}^{j}}.$$

Credible intervals that ensure 95% of concentration profiles are contained between the two bounds are shown in figure 1 using r=0.025 and r=0.975 in (A 8); the median is evaluated using r=0.5. Credible intervals respect the requirement that the concentration is bounded between $C_{N}$ at the outlet and $C_{0}$ at the inlet, demonstrating that the solute distribution is non-Gaussian.

## Appendix B. Moments of the sink distribution

Let sink locations in three dimensions be prescribed by a multivariate uniform distribution, with position vectors $\xi_{i_{3}}=(\xi_{i},\xi_{j},\xi_{k})$ such that $\xi_{i}∼U[0,1]$ and $\xi_{j},\xi_{k}∼U[-L_{s},L_{s}]$ for i=1,…,N and j,k=−M,…,M. Each continuous uniformly random variable $\xi_{i_{3}}$ is independently and identically distributed with a pdf given by

B 1 $$\pi_{\xi_{{\text{i}}_{3}}}({\text{x}}_{{\text{i}}_{3}})=\left\{ \begin{array}{ll} \frac{1}{{\left(2L_{s}\right)}^{2}}=\frac{1}{\rho^{2}{\left(2M+1\right)}^{2}} & \text{for }{\text{x}}_{{\text{i}}_{3}}\in D_{3}^{s},\\ 0 & \text{otherwise.} \end{array}\right.$$

Using the definition of the sink function given in (2.2), the expectation of $\hat{g}(x;\omega )$ is given by

B 2 $$\begin{array}{rl} E[\hat{g}(\text{x};\omega )] & \;=\int_{D_{3}}\int_{D_{3}}\ldots \left(\rho^{3}\sum_{{\text{i}}_{3}}F_{\varsigma }^{\left(3\right)}(\text{x}-{\text{x}}_{{\text{i}}_{3}})-1\right)\pi_{\xi_{\text{1}},\xi_{\text{2}},\ldots }({\text{x}}_{\text{1}},{\text{x}}_{\text{2}},\ldots )\;d{\text{x}}_{\text{1}}d{\text{x}}_{\text{2}}\ldots \\ & \;=\rho^{3}\sum_{{\text{i}}_{3}}\int_{D_{3}}F_{\varsigma }^{\left(3\right)}(\text{x}-{\text{x}}_{{\text{i}}_{3}})\pi_{\xi_{{\text{i}}_{3}}}({\text{x}}_{{\text{i}}_{3}})\;d{\text{x}}_{{\text{i}}_{3}}-1=0. \end{array}$$

To calculate the covariance $K_{\hat{g}}(x,y)=E[\hat{g}(x;\omega )\hat{g}(y;\omega )],$ we can again use (B 1) to obtain

$$\begin{array}{rl} K_{\hat{g}}(\text{x},\text{y}) & \;=\rho^{6}\underset{{\text{i}}_{3}\neq {\text{j}}_{3}}{\sum_{{\text{i}}_{3}}\sum_{{\text{j}}_{3}}}\int_{D_{3}}\int_{D_{3}}F_{\varsigma }^{\left(3\right)}(\text{x}-{\text{x}}_{{\text{i}}_{3}})F_{\varsigma }^{\left(3\right)}(\text{y}-{\text{x}}_{{\text{j}}_{3}})\pi_{\xi_{{\text{i}}_{3}},\xi_{{\text{j}}_{3}}}({\text{x}}_{{\text{i}}_{3}},{\text{x}}_{{\text{j}}_{3}})\;d{\text{x}}_{{\text{i}}_{3}}\;d{\text{x}}_{{\text{j}}_{3}}\\ & \;\;+\rho^{6}\sum_{{\text{i}}_{3}}\int_{D_{3}}F_{\varsigma }^{\left(3\right)}(\text{x}-{\text{x}}_{{\text{i}}_{3}})F_{\varsigma }^{\left(3\right)}(\text{y}-{\text{x}}_{{\text{i}}_{3}})\pi_{\xi_{{\text{i}}_{3}}}({\text{x}}_{{\text{i}}_{3}})\;d{\text{x}}_{{\text{i}}_{3}}\\ & \;\;-\rho^{3}\sum_{{\text{i}}_{3}}\int_{D_{3}}F_{\varsigma }^{\left(3\right)}(\text{x}-{\text{x}}_{{\text{i}}_{3}})\pi_{\xi_{{\text{i}}_{3}}}({\text{x}}_{{\text{i}}_{3}})\;d{\text{x}}_{{\text{i}}_{3}}-\rho^{3}\sum_{{\text{j}}_{3}}\int_{D_{3}}F_{\varsigma }^{\left(3\right)}(\text{y}-{\text{x}}_{{\text{j}}_{3}})\pi_{\xi_{{\text{j}}_{3}}}({\text{x}}_{{\text{j}}_{3}})\;d{\text{x}}_{{\text{j}}_{3}}+1, \end{array}$$

which gives

B 3 $$K_{\hat{g}}(\text{x},\text{y})=\rho^{3}F_{\varsigma }^{\left(3\right)}(\text{x},\text{y})-\frac{\rho }{{\left(2M+1\right)}^{2}},\;\mathrm{where}\;F_{\varsigma }^{\left(3\right)}(\text{x},\text{y})\equiv \int_{D_{3}}F_{\varsigma }^{\left(3\right)}(\text{x}-\hat{\text{x}})F_{\varsigma }^{\left(3\right)}(\text{y}-\hat{\text{x}})\;d\hat{\text{x}}.$$

The function $F_{\varsigma }^{\left(3\right)}$ measures the overlap of the two functions $F_{\varsigma }^{\left(3\right)}(x-\hat{x})$ and $F_{\varsigma }^{\left(3\right)}(y-\hat{x})$ and is zero when x is sufficiently far from y. When $F_{\varsigma }^{\left(3\right)}$ has a Gaussian structure (2.4), we find that

$$F_{\varsigma }^{\left(3\right)}(\text{x},\text{y})=I_{2}(x_{1},y_{1};\varsigma ,\varsigma )I_{2}(x_{2},y_{2};\varsigma ,\varsigma )I_{2}(x_{3},y_{3};\varsigma ,\varsigma )=F_{\sqrt{2}\varsigma }^{\left(3\right)}(\text{x}-\text{y}),$$

where

B 4 $$I_{2}(x,y;\sigma_{x},\sigma_{y})\equiv \frac{1}{2\pi \sigma_{x}\sigma_{y}}\int_{-\infty }^{\infty }\exp{\left(-\frac{1}{2\sigma_{x}^{2}}(\hat{x}-x\right)}^{2}-\frac{1}{2\sigma_{y}^{2}}{\left(\hat{x}-y\right)}^{2})d\hat{x}.$$

This in turn gives the covariance of $\hat{g}$ as in (2.5) for n=3; we can extend these results using similar calculations for n=1 and 2 dimensions, noting that the number of sinks ${\left(2M+1\right)}^{2}/\rho$ becomes ${\left(2M+1\right)}^{n-1}/\rho$.

## Appendix C. Green’s functions

The exact Green’s function in one-dimensional $G(x_{1},x_{1}^{\prime })$ satisfies $LG=\delta (x_{1}-x_{1}^{\prime })$, $B_{1}G=\{0,0\}$, where $L={\left(\partial_{x_{1}}\right)}^{2}-\mathrm{Pe}\partial_{x_{1}}-\mathrm{Da}$ and $B_{1}=\{(1-(1/\mathrm{Pe})\partial_{x_{1}})(\cdot ){|}_{x_{1}=0},\;\partial_{x_{1}}(\cdot ){|}_{x_{1}=1}\}$. We define $G^{-}(x_{1},x_{1}^{\prime })$ and $G^{+}(x_{1},x_{1}^{\prime })$ such that

$$G(x_{1},x_{1}^{\prime })=\left\{ \begin{array}{ll} G^{-}(x_{1},x_{1}^{\prime }) & \text{if }0\leq x_{1}\leq x_{1}^{\prime }\leq 1\\ G^{+}(x_{1},x_{1}^{\prime }) & \text{if }0\leq x_{1}^{\prime }\leq x_{1}\leq 1, \end{array}\right.$$

where

C 1 $$\begin{array}{rl} & G^{\pm }(x_{1},x_{1}^{\prime })=-\left(\frac{1}{4\phi \psi (1)}\right)\;e^{\left(\mathrm{Pe}/2)(x_{1}-x_{1}^{\prime }\right)}({\left(2\phi +\mathrm{Pe}\right)}^{2}\;e^{\phi (\pm (x_{1}^{\prime }-x_{1})+1)}\\ & \;\;+{\left(2\phi -\mathrm{Pe}\right)}^{2}\;e^{-\phi (\pm (x_{1}^{\prime }-x_{1})+1)}+4\mathrm{Da}(e^{\phi (x_{1}+x_{1}^{\prime }-1)}+\;e^{-\phi (x_{1}+x_{1}^{\prime }-1)})). \end{array}$$

It is convenient to re-express this to allow numerical evaluation when ${\mathrm{Pe}}^{2}\gg \mathrm{Da}$. We expand exponential terms to obtain

$$\phi \approx \frac{\mathrm{Pe}}{2}+\frac{\mathrm{Da}}{\mathrm{Pe}}-\frac{{\mathrm{Da}}^{2}}{{\mathrm{Pe}}^{3}},\;\exp(\pm \phi x_{1})\approx \left(1\mp \frac{{\mathrm{Da}}^{2}}{{\mathrm{Pe}}^{3}}x_{1}\right)\exp\left(\pm \left(\frac{\mathrm{Pe}}{2}+\frac{\mathrm{Da}}{\mathrm{Pe}}\right)x_{1}\right)$$

and

$$\psi (x_{1})\approx 2{\mathrm{Pe}}^{2}\left(1+\frac{\mathrm{Da}}{{\mathrm{Pe}}^{2}}\left(2-\frac{\mathrm{Da}}{\mathrm{Pe}}x_{1}\right)\right)\exp\left(\left(\frac{\mathrm{Pe}}{2}+\frac{\mathrm{Da}}{\mathrm{Pe}}\right)x_{1}\right),$$

giving

C 2 $$\begin{array}{rl} {\tilde{G}}^{-}(x_{1},x_{1}^{\prime }) & \;\approx -\frac{1}{\mathrm{Pe}}\;e^{\left(\mathrm{Pe}+(\mathrm{Da}/\mathrm{Pe}))(x_{1}-x_{1}^{\prime }\right)}\\ & \;\;+\frac{\mathrm{Da}}{{\mathrm{Pe}}^{3}}(\left(2+\frac{\mathrm{Da}}{\mathrm{Pe}}(x_{1}-x_{1}^{\prime })\right)\;e^{\left(\mathrm{Pe}+(\mathrm{Da}/\mathrm{Pe}))(x_{1}-x_{1}^{\prime }\right)}\\ & \;\;-e^{\mathrm{Pe}(x_{1}-1)+(\mathrm{Da}/\mathrm{Pe})(x_{1}+x_{1}^{\prime }-2)}-e^{-\mathrm{Pe}x_{1}^{\prime }-(\mathrm{Da}/\mathrm{Pe})(x_{1}+x_{1}^{\prime })})\\ \;\mathrm{and}\;{\tilde{G}}^{+}(x_{1},x_{1}^{\prime }) & \;\approx -\frac{1}{\mathrm{Pe}}\;e^{\left(\mathrm{Da}/\mathrm{Pe})(x_{1}^{\prime }-x_{1}\right)}\\ & \;\;+\frac{\mathrm{Da}}{{\mathrm{Pe}}^{3}}(\left(2+\frac{\mathrm{Da}}{\mathrm{Pe}}(x_{1}^{\prime }-x_{1})\right)e^{\left(\mathrm{Da}/\mathrm{Pe})(x_{1}^{\prime }-x_{1}\right)}\\ & \;\;-e^{\mathrm{Pe}(x_{1}-1)+(\mathrm{Da}/\mathrm{Pe})(x_{1}+x_{1}^{\prime }-2)}-e^{-\mathrm{Pe}x_{1}^{\prime }-(\mathrm{Da}/\mathrm{Pe})(x_{1}+x_{1}^{\prime })}). \end{array}\}$$

From the first term in ${\tilde{G}}^{-}\;({\tilde{G}}^{+}),$ we see a boundary layer of width approximately 1/Pe (Da⁡/Pe) exists upstream (downstream) of $x_{1}=x_{1}^{\prime }$ (figure 3*a*). The final two terms in ${\tilde{G}}^{-}$ and ${\tilde{G}}^{+}$ account for the boundary conditions, which gives a boundary layer of width approximately 1/Pe at the $x_{1}$-outlet and $x_{1}^{\prime }$-inlet.

The n-dimensional free-space Green’s function $G_{n}(x-x^{\prime })$ associated with (2.1) satisfies $L_{n}G^{n}=\delta (x-x^{\prime })$ and decays in the far field. Seeking a solution of the form $G_{n}(x)=\;{\text{e}}^{\lambda x_{1}}f(r)$ where r=|x| and setting λ=Pe⁡/2 leads to

C 3 $$G_{n}(\text{x}-{\text{x}}^{\prime })=-{\left(2\pi \right)}^{-n/2}{\left(\frac{\phi }{|\text{x}-{\text{x}}^{\prime }|}\right)}^{n/2-1}K_{n/2-1}(\phi |\text{x}-{\text{x}}^{\prime }|)\exp\left(\frac{\mathrm{Pe}}{2}(x_{1}-x_{1}^{\prime })\right),$$

where $\phi \equiv \sqrt{{\text{Pe}}^{2}/4+\text{Da}}$ and $K_{\nu }$ represents the modified Bessel function of the second kind [30]. The free-space Green’s function in one dimension is readily evaluated, noting that $K_{\pm 1/2}(z)=\sqrt{\pi /\left(2z\right)}\exp(-z)$, as

C 4 $$G_{1}(x_{1}-x_{1}^{\prime })=-\frac{1}{2\phi }\exp\left(\frac{\mathrm{Pe}}{2}(x_{1}-x_{1}^{\prime })-\phi |x_{1}-x_{1}^{\prime }|\right).$$

As illustrated in figure 3*a*,*b*, for $\text{Pe}\gg \max(1,\sqrt{\text{Da}})$, $G_{1}$ decays on a short lengthscale 1/Pe upstream of $x_{1}=x_{1}^{\prime }$, and on a long lengthscale Pe⁡/Da downstream, but fails to capture additional boundary layers of width 1/Pe in $G^{+}$ at the edges of the domain.

From (C 3), the two-dimensional free-space Green’s function is

C 5 $$G_{2}(\text{x}-{\text{x}}^{\prime })=-\frac{1}{2\pi }K_{0}(\phi |\text{x}-{\text{x}}^{\prime }|)\exp\left(\frac{\mathrm{Pe}}{2}(x_{1}-x_{1}^{\prime })\right).$$

Thus

C 6 $$G_{2}(\text{x}-{\text{x}}^{\prime })\approx \left\{ \begin{array}{ll} \frac{1}{2\pi }\log(\phi |\text{x}-{\text{x}}^{\prime }|) & \phi |\text{x}-{\text{x}}^{\prime }|\ll 1,\\ -\frac{1}{2}\sqrt{\frac{1}{2\pi \phi |\text{x}-{\text{x}}^{\prime }|}}\exp\left(\frac{\mathrm{Pe}}{2}(x_{1}-x_{1}^{\prime })-\phi |\text{x}-{\text{x}}^{\prime }|\right) & \phi |\text{x}-{\text{x}}^{\prime }|\gg 1. \end{array}\right.$$

Along $x_{2}=x_{2}^{\prime }$, when Pe≫max(1,Da), $G_{2}$ decays over the same lengthscales as $G_{1}$. Along $x_{1}=x_{1}^{\prime }$, $G_{2}$ decays over a distance 1/Pe in the $x_{2}$ direction. The asymptotic shape of the wake in the far field is revealed by rescaling using $x_{1}-x_{1}^{\prime }=(\text{Pe}/\text{Da})X_{1}$ and $x_{2}-x_{2}^{\prime }=(1/\sqrt{\text{Da}})X_{2}$ for $X_{1},X_{2}=O(1)$. We can then approximate (C 6*b*) as

C 7 $$G_{2}(\text{x}-{\text{x}}^{\prime })\approx -\frac{1}{2\mathrm{Pe}}\sqrt{\frac{\mathrm{Da}}{\pi X_{1}}}\exp\left(-X_{1}-\frac{X_{2}^{2}}{4X_{1}}+\ldots \right),$$

for $\text{Pe}\gg \sqrt{\text{Da}}$. The argument of the exponential identifies the approximately elliptical shape of concentration contours, as sketched in figure 3*c*.

The three-dimensional free-space Green’s function is

C 8 $$G_{3}(\text{x}-{\text{x}}^{\prime })=-\frac{1}{4\pi |\text{x}-{\text{x}}^{\prime }|}\exp\left(\frac{\mathrm{Pe}}{2}(x_{1}-x_{1}^{\prime })-\phi |\text{x}-{\text{x}}^{\prime }|\right).$$

This has near-field form

C 9 $$G_{3}(\text{x}-{\text{x}}^{\prime })\approx -\frac{1}{4\pi r},\;\mathrm{as}\;r=|\text{x}-{\text{x}}^{\prime }|\to 0$$

while the far-field structure for $\text{Pe}\gg \max(1,\sqrt{\text{Da}})$ can be written

C 10 $$G_{3}(\text{x}-{\text{x}}^{\prime })\approx -\frac{1}{4\pi (x_{1}-x_{1}^{\prime })}\exp\left[-\frac{\mathrm{Da}}{\mathrm{Pe}}(x_{1}-x_{1}^{\prime })-\mathrm{Pe}\frac{{\left(x_{2}-x_{2}^{\prime }\right)}^{2}+{\left(x_{3}-x_{3}^{\prime }\right)}^{2})}{4(x_{1}-x_{1}^{\prime })}\right],$$

with lengthscales resembling those illustrated in figure 3*c*.

## Appendix D. Evaluating integrals

In one dimension, (C 4) with $x_{1}^{\prime \prime }=x_{1}^{\prime }+\varsigma u$ gives $G_{1}(x_{1}^{\prime }-x_{1}^{\prime \prime })=-(1/\left(2\phi \right))\exp(-\frac{1}{2}\text{Pe}\varsigma u-\phi \varsigma |u|)$. Therefore,

$$\begin{array}{rl} & \;\int_{D_{1}}G_{1}(x_{1}^{\prime }-x_{1}^{\prime \prime })C_{H}(x_{1}^{\prime \prime })F_{\sqrt{2}\varsigma }^{\left(1\right)}(x_{1}^{\prime }-x_{1}^{\prime \prime })\;dx_{1}^{\prime \prime }\\ & \;\approx -\frac{1}{4\sqrt{\pi }\phi }C_{H}(x_{1}^{\prime })\int_{-\infty }^{\infty }\exp\left(-\frac{u^{2}}{4}-\frac{\mathrm{Pe}}{2}\varsigma u-\phi \varsigma |u|\right)\;du. \end{array}$$

The integral asymptotes to $2\sqrt{\pi }$ as ς→0, and we obtain $\beta_{1}=1/(2\phi )$ in (2.17). In two dimensions, (C 6*a*) with $x^{\prime \prime }=x^{\prime }+\varsigma u$ and $\hat{r}=|u|$ gives $G_{2}(x^{\prime }-x^{\prime \prime })=G_{2}(-\varsigma u)\approx (1/(2\pi ))\log(\phi \varsigma \hat{r})$ when ς≪1/ϕ≪1. Therefore,

$$\int_{D_{2}}G_{2}({\text{x}}^{\prime }-{\text{x}}^{\prime \prime })C_{H}(x_{1}^{\prime \prime })F_{\sqrt{2}\varsigma }^{\left(2\right)}({\text{x}}^{\prime }-{\text{x}}^{\prime \prime })\;d{\text{x}}^{\prime \prime }\approx \frac{1}{4\pi }C_{H}(x_{1}^{\prime \prime })\int_{0}^{\infty }\hat{r}\log(\phi \varsigma \hat{r})\exp(-\frac{{\hat{r}}^{2}}{4})\;d\hat{r}.$$

The integral is evaluated using the identity

D 1 $$\int_{0}^{\infty }x\log(bx)\exp(-ax^{2})dx=\frac{1}{2a}\log\left(\frac{b}{\sqrt{a}}\right)+\frac{\gamma }{4a}$$

(using Van Heemert [31], where γ≈0.577 is the Euler–Mascheroni constant), to obtain $\beta_{2}=(\gamma -2\log(2\phi \varsigma ))/(4\pi )$ in (2.17). In three dimensions, (C 9) with $x^{\prime \prime }=x^{\prime }+\varsigma u$ and $\hat{r}=|u|$ gives $G_{3}(x^{\prime }-x^{\prime \prime })=G_{3}(-\varsigma u)\approx -1/(4\pi \varsigma \hat{r})$ when ς≪1/ϕ≪1. Therefore,

$$\int_{D_{3}}G_{3}({\text{x}}^{\prime }-{\text{x}}^{\prime \prime })C_{H}(x_{1}^{\prime \prime })F_{\sqrt{2}\varsigma }^{\left(3\right)}({\text{x}}^{\prime }-{\text{x}}^{\prime \prime })\;d{\text{x}}^{\prime \prime }\approx -\frac{1}{{\left(4\pi \right)}^{3/2}\varsigma }C_{H}(x_{1}^{\prime \prime })\int_{0}^{\infty }\hat{r}\exp(-\frac{{\hat{r}}^{2}}{4})\;d\hat{r},$$

giving $\beta_{3}=1/4\pi^{3/2}\varsigma$ in (2.17).

Integrals involving the one-dimensional Green’s function convolved with $C_{H}$ can be evaluated exactly when the free-space function $G_{1}$ is used. These can be simplified by eliminating terms that are exponentially small throughout the domain, when Pe≫1. The resulting expressions are

D 2 $$\int_{D_{1}}G_{1}(x_{1}-x_{1}^{\prime })C_{H}(x_{1}^{\prime })\;dx_{1}^{\prime }\approx \frac{\mathrm{Pe}\;e^{(\mathrm{Pe}/2)x_{1}}}{4\phi^{2}\hat{\psi }(1)}(2\mathrm{Pe}\;e^{\phi (x_{1}-1)}-(2\phi +\mathrm{Pe})(1+2\phi x_{1})e^{\phi (1-x_{1})}),$$

D 3 $$\begin{array}{rl} & \;\int_{D_{1}}\int_{D_{1}}G_{1}(x_{1}-x_{1}^{\prime })G_{1}(x_{1}^{\prime }-x_{1}^{\prime \prime })C_{H}(x_{1}^{\prime \prime })\;dx_{1}^{\prime }\;dx_{1}^{\prime \prime }\\ & \;\approx \frac{\mathrm{Pe}\;e^{(\mathrm{Pe}/2)x_{1}}}{8\phi^{4}\hat{\psi }(1)}((2\phi +\mathrm{Pe}){\left(1+\phi x_{1}\right)}^{2}\;e^{\phi (1-x_{1})}+\left(-\frac{5\mathrm{Pe}}{2}-\phi (1+\mathrm{Pe}+2\phi )\right)\;e^{\phi (x_{1}-1)}) \end{array}$$

and

D 4 $$\begin{array}{rl} & \;\int_{D_{1}}{\left[G_{1}(x_{1}-x_{1}^{\prime })C_{H}(x_{1}^{\prime })\right]}^{2}\;dx_{1}^{\prime }\\ & \;\approx \left(\frac{{\mathrm{Pe}}^{2}}{16\phi^{3}\hat{\psi }{\left(1\right)}^{2}}\right)e^{\mathrm{Pe}x_{1}}({\left(2\phi +\mathrm{Pe}\right)}^{2}(4\phi x_{1}+1)\;e^{2\phi (1-x_{1})}\\ & \;\;+4(4\phi^{2}-{\mathrm{Pe}}^{2})(2-\;e^{-2\phi x_{1}})-4(4\phi^{2}+2\phi \mathrm{Pe}-{\mathrm{Pe}}^{2})\;e^{2\phi (x_{1}-1)}), \end{array}$$

with $\hat{\psi }$ being the approximation of ψ near $x_{1}=1$, which is given by $\hat{\psi }(x_{1})=(2\text{Pe}\phi +{\text{Pe}}^{2}+2\text{Da})\;{\text{e}}^{\phi x_{1}}$.

## Appendix E. Integrals for effective uptake

Consider a Gaussian covariance function of the form $\hat{K}(x-y)=\sigma^{2}\exp(-|x-y{|}^{2}/\ell^{2})$. Then (2.27) gives

$$G_{2}\hat{K}(\text{0})\approx -\frac{\sigma^{2}}{2\pi }\int_{R^{2}}\exp\left(\frac{\mathrm{Pe}}{2}x_{1}-\frac{|\text{x}{|}^{2}}{\ell^{2}}\right)K_{0}(\phi |\text{x}|)\;d\text{x}.$$

By converting to polar coordinates where $x_{1}=r\cos\theta$ and $x_{2}=r\sin\theta$, we can solve the θ integral by using

E 1 $$\int_{0}^{2\pi }\exp(z\cos\theta )\;d\theta =2\pi I_{0}(z),$$

where $I_{\nu }$ is a modified Bessel function of the first kind [32], to give

$$G_{2}\hat{K}(\text{0})\approx -\sigma^{2}\int_{0}^{\infty }\exp\left(-\frac{r^{2}}{\ell^{2}}\right)I_{0}\left(\frac{\mathrm{Pe}}{2}r\right)K_{0}(\phi r)r\;dr.$$

We set r=ℓR and approximate the Bessel functions using

E 2 $$I_{0}\left(\left(\frac{\mathrm{Pe}}{2}\right)\ell R\right)\approx 1+O(l^{2}{\mathrm{Pe}}^{2}R^{2}),\;K_{0}(\phi \ell R)\approx -\log(\phi \ell R)=-\log(\phi \ell )-\log(R)\;\mathrm{as}\;\ell \to 0,$$

to give

$$G_{2}\hat{K}(\text{0})\approx \sigma^{2}\ell^{2}\left(\log(\phi \ell )\int_{0}^{\infty }R\exp(-R^{2})\;dR+\int_{0}^{\infty }R\log R\exp(-R^{2})\;dR\right),$$

for suitably small ℓ. Using (D 1), we obtain $G_{2}\hat{K}(0)\approx -\frac{1}{4}\sigma^{2}\ell^{2}(\gamma -2\log(\phi \ell ))$, hence yielding the two-dimensional result in (2.29). The analogous integral in three dimensions reduces to

$$G_{3}\hat{K}(\text{0})\approx -\frac{\sigma^{2}}{2}\int_{0}^{\pi }\int_{0}^{\infty }\exp\left(-\phi r-\frac{r^{2}}{\ell^{2}}\right)I_{0}\left(\frac{\mathrm{Pe}}{2}r\sin\theta \right)r\sin\theta \;dr\;d\theta .$$

Again for small ℓ, we use (E 2) to evaluate the integral for r=O(ℓ), leading to $G_{3}\hat{K}(0)\approx -\frac{1}{2}\sigma^{2}\ell^{2}$, the three-dimensional limit in (2.29).
